# Optimization of callus induction and proliferation of *Paeonia lactiflora* Pall. and *Agrobacterium*-mediated genetic transformation

**DOI:** 10.3389/fpls.2022.996690

**Published:** 2022-12-16

**Authors:** Siyang Duan, Rujie Xin, Shixin Guan, Xueting Li, Riwen Fei, Wan Cheng, Qing Pan, Xiaomei Sun

**Affiliations:** ^1^ College of Forestry, Shenyang Agricultural University, Shenyang, China; ^2^ Key Laboratory of Forest Tree Genetics, Breeding and Cultivation of Liaoning Province, Shenyang Agricultural University, Shenyang, China; ^3^ College of Horticulture, Shenyang Agricultural University, Shenyang, China

**Keywords:** herbaceous peony, callus, induction, proliferation, *Agrobacterium*, genetic transformation, GUS expression

## Abstract

*Paeonia lactiflora* Pall. is an important ornamental plant with high economic and medicinal value, which has considerable development prospects worldwide. The lack of efficient tissue culture techniques and genetic transformation systems has become a master obstacle for *P. lactiflora* research. The purpose of the present study focuses on obtaining an efficient and stable genetic transformation method using callus as the receptor and exploring an efficient protocol for callus induction and proliferation associated with *P. lactiflora*. Callus induction and proliferation were performed using MS medium with various concentrations of 2,4-Dichlorophenoxyacetic acid (2,4-D), 1-Naphthaleneacetic acid (NAA), 6-Benzylaminopurine (6-BA) and thidiazuron (TDZ). The sensitivity of callus to kanamycin and cefotaxime was determined. Several parameters such as *Agrobacterium* cell density, infection time and co-culture duration were studied to optimize transformation efficiency. *Agrobacterium* strains EHA105 and pBI121 binary vector harboring the *β-glucuronidase* (GUS) gene were used for transformation. Expression of the GUS reporter gene was detected by GUS assay, polymerase chain reaction (PCR) and Quantitative Real-time PCR (RT-qPCR). The MS medium containing 1.0 mg·L^-1^ NAA, 0.5 mg·L^-1^ 2,4-D and 0.5 mg·L^-1^ TDZ was optimal for callus induction and MS medium containing 0.5 mg·L^-1^ NAA, 1.0 mg·L^-1^ 2,4-D and 0.5 mg·L^-1^ TDZ was the best for callus proliferation. The concentrations of kanamycin and cefotaxime used for screening positive callus were 125 mg·L^-1^ and 200 mg·L^-1^, respectively. Among various combinations analyzed, the best transformation result was obtained *via* the 25 min of infection of *Agrobacterium* at 0.6 OD_600_ and 3 d of co-culture. Overall, this study provided technical support and theoretical guidance for improving the callus induction and proliferation efficiency and the study of gene function in *P. lactiflora*.

## Introduction

Herbaceous peony (*Paeonia lactiflora* Pall.) is a perennial herbaceous plant of the genus *Paeonia* (Zhang et al., 2022). Because of its large, colorful and diverse flowers, herbaceous peony is used as garden, potted, cut and dried flowers in many countries and regions (Zhao et al., 2017; Liu et al., 2022). In addition, it is also used as an important source of crude drugs in traditional Chinese medicine (Hu et al., 2015; Sun et al., 2021). Due to its high ornamental, medicinal and economic value, herbaceous peony is widely cultivated in the world (Xue et al., 2018; Zhao et al., 2019).

Seed and dividing are the traditional propagation methods of herbaceous peony. However, due to the special epicotyl and hypocotyl double dormancy characteristics of herbaceous peony seeds, it usually takes 3-5 years from seed germination to flowering, which makes it difficult for traditional breeding methods to meet the needs of commercial production (Shen et al., 2014). Finding efficient propagation methods of herbaceous peony has become a significant and meaningful research task. In recent years, there is an increasing and new interest to plant tissue culture (Georgiev et al., 2018). Tissue culture can increase the propagation rate, and is an important way to rapidly propagate plants, improve popular plant varieties or select new plant varieties (Osakabe et al., 2018). Although various studies have been studied on vitro culture of herbaceous peony (Jana et al., 2013; Shen et al., 2014; Zhao et al., 2017), many problems continue to be encountered on establishing faster and more efficient methods of tissue culture and its large-scale production. Numerous hurdles related to the tissue culture, such as low efficiency in direct or indirect induction of adventitious shoots, high levels of contamination, browning, poor rooting, and difficulties in transplanting and acclimatization, still need to be solved (Sivanesan et al., 2021). Therefore, it is necessary to establish an efficient and stable method of callus induction and proliferation to alleviate or even solve the above problems, and to establish a basis for the establishment of stable regeneration system of herbaceous peony.

Transgenic plants are commonly used in molecular biology to understand the function of specific genes( Chakraborty et al., 2020). The establishment of an efficient, stable and regenerating receptor system is the basis for genetic transformation. However, there are still so many obstacles on the establishment of the regeneration system associated with herbaceous peony, which not only affect the tissue culture, but also hinder the molecular biology research. Due to the difficulty in regeneration of herbaceous peony during tissue culture, there is no stable genetic transformation system and transgenic plants cannot be obtained, which greatly limits the transgenic work of herbaceous peony (Wang et al., 2021a). Therefore, at present, studies on gene function verification of *P. lactiflora* are carried out either through heterologous transformation or homologous transient expression (Ni et al., 2018; Bai et al., 2019; Lv et al., 2019). Based on this situation, finding other transgenic receptors to carry out molecular biological research of herbaceous peony has been the focus of scholars (Shen et al., 2012). Gene transformation using callus as receptor has the advantages of high efficiency and short transformation time, and has been widely used in functional analysis of related genes in apple, citrus, grape and other plants (Wu et al., 2017; Xu et al., 2017; Osakabe et al., 2018). Callus of herbaceous peony is easy to obtain, has strong proliferation ability, and can also be used as receptor material for transgene (Guo and Jeong, 2021). At the same time, with the rapid development of plant genetic transformation research, *Agrobacterium*-mediated transformation has become a convenient tool to transfer favorable traits to plants, and has already successfully applied to many plants (Chetty et al., 2013; Mayavan et al., 2013; Chen et al., 2014; Palla and Pijut, 2014; Liu et al., 2015; Singh et al., 2016; Sedaghati et al., 2018; Sun et al., 2019). However, there are few reports on *Agrobacterium*-mediated genetic transformation using herbaceous peony callus as receptor. By transferring genes into callus, the function and molecular mechanism of many important genes in *P. lactiflora* can be determined (An et al., 2018; Qi et al., 2018; Wang et al., 2018; Lu et al., 2019; Zhao et al., 2019b).

To help solve the above problems, in this study, we optimized the callus induction and proliferation protocol of *P. lactiflora* and further established an efficient *Agrobacterium*-mediated transgenic method with callus as receptor. We believe that our results provide a theoretical basis and technical support for the establishment of tissue culture regeneration system and further verification of gene function of herbaceous peony.

## Materials and methods

### Plant material and treatment

Herbaceous peony hybrid seeds (‘Fen Yu Nu’×’Xi Shi Fen’) were harvested in the germplasm resource nursery of Shenyang Agricultural University, Liaoning, China. Full and large seeds were chosen and washed under the running tap water for 10 min. After soaking in distilled water for 48 h, the seed coat was removed. Subsequently, they were transferred to a clean bench for further sterilization. The seeds were dipped in 75% (v/v) ethanol for 30 s, and rinsed 2 times with sterile distilled water. Followed by 15 min sterilization with sodium hypochlorite solution (1.5%, w/v), and the seeds were finally rinsed three times in sterile distilled water. After sterilization, the much of endosperm in each seed was removed to leave isolated embryo with a bit of endosperm, which was cultured on Murashige and Skoog (MS) medium supplemented with 0.5 mg·L^-1^ GA_3_ (initiation medium) for 45 d to obtain sterile seedlings. All medium and agar used in this article were purchased from Beijing Solarbio Science & Technology Co., LTD. All plant growth regulators (PGRs) were purchased from Shanghai Yien Chemical Technology Co., LTD.

### Culture and growing conditions

The culture vessels were 100 mL Erlenmeyer flasks with 30 mL of medium. Plants were grown in a tissue culture room at 25 ± 2°C with a 16 h light/8 h dark photoperiod in 50 μmol·m^−2^·s^−1^ photosynthetic photon flux density of cool white fluorescent tubes.

### Callus induction

The epicotyl stems were cut into 8-10 mm lengths, and both the leaves and cotyledons were cut into 8 mm × 8 mm squares. They were both transferred to MS medium supplemented PGRs, including 0.5 mg·L^-1^ 6-Benzyleaminopurine (6-BA), 1.0 mg·L^-1^ 1-Naphthylacetic acid (NAA) and 1.0 mg·L^-1^ 2,4-dichlorophenoxyacetic acid (2,4-D). Since cotyledons had the best callus induction effect and were easy to obtain, the effects of different PGRs on callus induction were studied by taking sterile cotyledons as explants. According to the results of the preliminary experiment, they were transferred to MS medium containing various concentrations of PGRs alone or in combination, including 6-BA (0.1 mg·L^-1^, 0.5 mg·L^-1^, 1.0 mg·L^-1^), 2,4-D (0.1 mg·L^-1^, 0.5 mg·L^-1^, 1.0 mg·L^-1^), NAA (0.1 mg·L^-1^, 0.5 mg·L^-1^, 1.0 mg·L^-1^) and Thidiazuron (TDZ) (0.5 mg·L^-1^, 1.0 mg·L^-1^, 2.0 mg·L^-1^) (**Table 1**). Each of the treatments was conducted with at least 30 explants. The callus induction rate was calculated and the callus growth state was observed after 30 d of culture.

**Table 1:** Effect of 2.4-D (mg·L_-1_), NAA, TDZ and 6-BA on *P. lactiflora* callus induction.

Groups	2, 4-D (mg·L^-1^)	NAA (mg·L^-1^)	TDZ (mg·L^-1^)	6-BA (mg·L^-1^)	Induction rate (%)	Growth state
	0.1	–	–	–	41.11 ± 1.92c	emerald-green and loose-crunchy
1	0.5	–	–	–	50.00 ± 3.33b	emerald-green and tight-dense
	1.0	–	–	–	57.78 ± 5.09a	light-green and tight-dense
	–	0.1	–	–	46.67 ± 3.33b	yellow-white and loose
2	–	0.5	–	–	54.44 ± 1.92a	yellow-white and loose
	–	1.0	–	–	41.11 ± 1.92c	yellow-white and loose
	1.0	–	0.5	–	23.33 ± 5.78b	emerald-green and loose-crunchy
3	1.0	–	1.0	–	67.78 ± 1.92a	emerald-green and loose-crunchy
	1.0	–	2.0	–	26.67 ± 3.33b	emerald-green and loose-crunchy
	1.0	–	–	0.5	44.44 ± 1.92b	yellow-green and tight-dense
4	1.0	–	–	1.0	57.78 ± 1.92 a	yellow-green and tight-dense
	1.0	–	–	2.0	53.33 ± 3.33 a	yellow-green and tight-dense
	0.1	0.1	0.5	–	58.89 ± 1.82 c	emerald-green and tight-dense
	0.1	0.5	1.0	–	52.22 ± 1.92 d	emerald-green and tight-dense
	0.1	1.0	2.0	–	46.67 ± 5.78 e	green and tight-dense
	0.5	0.1	1.0	–	62.22 ± 1.92 c	emerald-green and tight-dense
5	0.5	0.5	2.0	–	78.89 ± 1.92 b	emerald-green and tight-dense
	0.5	1.0	0.5	–	86.67 ± 3.33 a	green and tight-dense
	1.0	0.1	2.0	–	76.67 ± 3.33 b	emerald-green and tight-dense
	1.0	0.5	0.5	–	86.67 ± 3.33a	emerald-green and tight-dense
	1.0	1.0	1.0	–	81.11 ± 1.92b	green and tight-dense

Statistical analysis performed within each group. The values represented mean ± SD, and different letters within a column marked significant differences (p < 0.05).

### Callus proliferation

Callus from the sterile stem, leave and cotyledon were transferred respectively to MS medium containing 1.0 mg·L^-1^ TDZ, 1.0 mg·L^-1^ NAA and 0.5 mg·L^-1^ 2,4-D to observe their proliferation abilities. For the best proliferation effect, the effects of different PGRs on callus proliferation were studied by taking sterile cotyledons as explants. They were transferred to MS medium containing 2,4-D (0.1 mg·L^-1^, 0.5 mg·L^-1^, 1.0 mg·L^-1^), NAA (0.1 mg·L^-1^, 0.5 mg·L^-1^, 1.0 mg·L^-1^) and TDZ (0.5 mg·L^-1^, 1.0 mg·L^-1^, 2.0 mg·L^-1^) alone or in combination (**Table 2**). There were at least 30 calluses in each treatment. The callus proliferation coefficient and browning rate were calculated after 30 d of culture.

**Table 2:** Effects of NAA, 2, 4-D and TDZ on *P. lactiflora* callus proliferation.

Groups	NAA	2,4-D	TDZ	Proliferation coefficient	Browning rate (%)
	–	0.1	0.5	1.96 ± 0.10a	25.56 ± 3.85b
1	–	0.5	0.5	2.05 ± 0.10a	22.22 ± 3.85b
	–	1.0	0.5	1.95± 0.11a	33.33 ± 3.33a
	0.1	–	0.5	1.56 ± 0.03b	34.44 ± 6.94a
2	0.5	–	0.5	1.68 ± 0.04a	23.33 ± 3.33b
	1.0	–	0.5	1.47 ± 0.17b	40.00 ± 5.77a
	0.1	0.1	0.5	1.85 ± 0.04e	27.78 ± 1.92cd
	0.1	0.5	1.0	1.76 ± 0.04f	22.22 ± 3.85de
	0.1	1.0	2.0	1.79 ± 0.03ef	34.44 ± 1.92b
	0.5	0.1	1.0	1.56 ± 0.02g	32.22 ± 3.84bc
3	0.5	0.5	2.0	1.97 ± 0.06d	24.44 ± 1.92de
	0.5	1.0	0.5	3.21 ± 0.06a	33.33 ± 5.78bc
	1.0	0.1	2.0	1.78 ± 0.04ef	18.89 ± 3.85e
	1.0	0.5	0.5	2.80 ± 0.04b	41.11 ± 1.92a
	1.0	1.0	1.0	2.07 ± 0.03c	41.11 ± 1.92a

Plant growth regulators unit: mg·L^-1^. Statistical analysis performed within each group. The values represented mean ± SD, and different letters within a column marked significant differences (p < 0.05).

### Antibiotic sensitivity assay of *P. lactiflora* callus

The tolerance limits of cotyledone-induced callus to antibiotic kanamycin (Kan) and cefotaxime (Cef) were assessed prior to *Agrobacterium*-mediated transformation. The Kan and Cef were sterilized by filter and added to the medium when the medium temperature cooled down to about 40°C. The callus was cultured on a proliferation medium supplemented with different concentrations of Kan (0, 75, 100, 125 and 150 mg·L^-1^) or Cef (0, 100, 200, 300 and 400 mg·L^-1^) to determine the concentration of antibiotic needed for the effective selection of transgenic callus. The callus growth state was observed after 14 d of culture. The Kan and Cef were purchased from Beijing Solarbio Science & Technology Co., LTD.

### Inhibition test of cefotaxime against *Agrobacterium*

The 20 μL of overnight *Agrobacterium tumefaciens* EHA105 culture was added to a liquid Luria Bertani (LB) medium containing different concentrations of Cef (0, 100, 200, 300 and 400 mg·L^-1^) with three biological replicates. After 48 h of incubation, the OD_600_ value was determined by ultraviolet (UV) spectrophotometer (Qingdao Juchuang Environmental Protection Group Co. LTD, Qingdao, China).

### Optimization of *Agrobacterium*-mediated transformation factors

The binary vector pBI121-GUS purchased from Shanghai Maokang Biotechnology Co., LTD. was transformed into the *Agrobacterium* strain EHA105 purchased from Shanghai Angyu Technology Co., LTD. 1000 ng pBI121-GUS vector plasmid was added to 100 μl of *Agrobacterium* EHA105 competent cells and gently mixed, then placed in ice, liquid nitrogen and water at 28°C for 5 min, and finally another ice bath for 5 min. 700 μl LB liquid medium was added to the above products and incubated for 3 h in an incubator at 28°C by shaking. After the shock culture, the bacteria were dipped and coated on solid LB medium containing 50 mg·L^-1^ Kan and incubated upside down for 2 days at 28°C. Single colonies on the above medium were selected for PCR validation to obtain positive strains. Then a positive transformant was picked and inoculated overnight in 2 mL fresh liquid LB medium containing 50 mg·L^-1^ rifampicin and 50 mg·L^-1^ Kan at 28 °C in 200×g. The overnight culture liquid was transferred to a 50 ml liquid LB medium at a ratio of 1:50 and then cultured until the *Agrobacterium* cell density (OD_600_) reached 0.2-1.0, and then the culture was centrifuged at 5000×g for 10 min. The resuspension solution containing 5% sucrose and 100 μM filter-sterilized acetosyringone was used to resuspend the *Agrobacterium* as the infection solution. The healthy callus was soaked in the above prepared infection solution for infection, and the callus was gently shaken to make it fully infected. After infection, the callus was transferred to sterile filter paper until almost dry, and then transferred to the co-culture medium (MS medium containing 0.5 mg·L^-1^ NAA, 1.0 mg·L^-1^ 2,4-D and 0.5 mg·L^-1^TDZ), dark culture was carried out at 28 °C. After three times of rinse in sterile water containing Cef of 200 mg·L^-1^ and blotting on sterile filter paper, the calluses were cultured on MS medium containing 0.5 mg·L^-1^ NAA, 1.0 mg·L^-1^ 2,4-D, 0.5 mg·L^-1^ TDZ and 200 mg·L^-1^ Cef. Again, three times of rinse in sterile water containing Cef of 200 mg L^-1^ and blotting on sterile filter paper, the calluses were transferred to a selection medium (MS medium containing 0.5 mg·L^-1^ NAA, 1.0 mg·L^-1^ 2,4-D, 0.5 mg·L^-1^ TDZ and 125 mg·L^-1^ Kan). After 49 d of culture, part of the resistant callus was taken for GUS staining and Molecular identification. To improve transformation efficiency, the key factors affecting transformation efficiency were optimized, including *Agrobacterium* cell density (0.2, 0.4, 0.6, 0.8 and 1.0 OD_600_), infection duration (10, 15, 20, 25 and 30 min) and co-culture duration (0, 1, 2, 3 and 4 d).

### GUS histochemical assay

GUS histochemical assay was conducted according to the described procedure (Jefferson, 1987). The resistant callus was divided into two parts, one part was stained with GUS solution, and wild-type callus was used as negative control. The staining results were observed and counted. The other part was used for molecular identification.

### Molecular identification of transgenic callus

Genomic DNA was extracted from the transformed and non-transformed callus using an extraction kit (Aidlab, Beijing, China). The sequence of primers used for *GUS* gene (1182bp): forward primer(5’-ATACCGAAAGGTTGGGCAGG-3’), and reverse primer(5’-ATAACGGTTCAGGCACAGCA-3’). The PCR procedure were as followed: pre-denaturation at 94°C for 3 min, followed by 35 cycles at 94°C for 30 s, 56°C for 30 s, and 72°C for 1 min, and final extension at 72°C for 5 min. The amplified products were analyzed by electrophoresis in 1.2% (w/v) agarose gels. Total RNAs of callus were extracted by RNAprep pure Plant Kit (TianGen, Beijing, China). The cDNAs were synthesized by PrimeScript™ RT Master Mix kit (Perfect Real Time) (Takara, Beijing, China). The RT-qPCR primers were designed with the Primer Premier 5.0 software based on the GUS gene: forward primer (5’-AAGAGCGATGAGCACGAGTA-3’), and reverse primer (5’-AGAAATCAAACAACCCACGA-3’). *PlACTIN* (GenBank accession no. JN105299.1) was used as an internal control (Li et al., 2020). The Actin genes were detected using primers as followed: forward primer (5’-GGTCTATTCTTGCTTCCCTC-3’) and reverse primer (5’-CCCTCTGCGTCTACACTTTC-3’). The RT-qPCR was performed with StepOne™ 7500 Real-Time PCR using the TB Green ^®^ Premix Ex Taq™ II (Tli RNaseH Plus) (Takara, Beijing, China). The reactions were accomplished according to the following method, holding stage: 95°C for 30 s; cycling stage: 40 cycles of 95°C for 5 s, 60°C for 30 s; melt curve stage: 95°C for 15 s, 60°C for 1 min, 95°C for 15 s. Each experiment was performed with three biological and technical replicates. The results were analyzed according to the 2^−ΔΔCt^ methods, and exhibited by GraphPad Prism 8.0.

### Statistical analysis

Three biological replicates were performed for all experimental treatments in this article. We used SPSS22.0 software to perform one-way analysis of variance (ANOVA) and Duncan’s method for multiple comparisons of the obtained data to determine whether the differences between the data were significant. The important data involved in the paper are calculated as follows: Induction rate = the number of inducted explants/total explants × 100%, Mortality rate = number of dead explants/total explants × 100%, Proliferation coefficient = weight of fresh proliferated callus/weight of inoculated callus, Browning rate = number of browned explants/total explants × 100%, GUS expression rate = the number of GUS-positive receptors/total number stained × 100%.

## Results

### Effect of different explant on inducing callus

**Figure 1:**
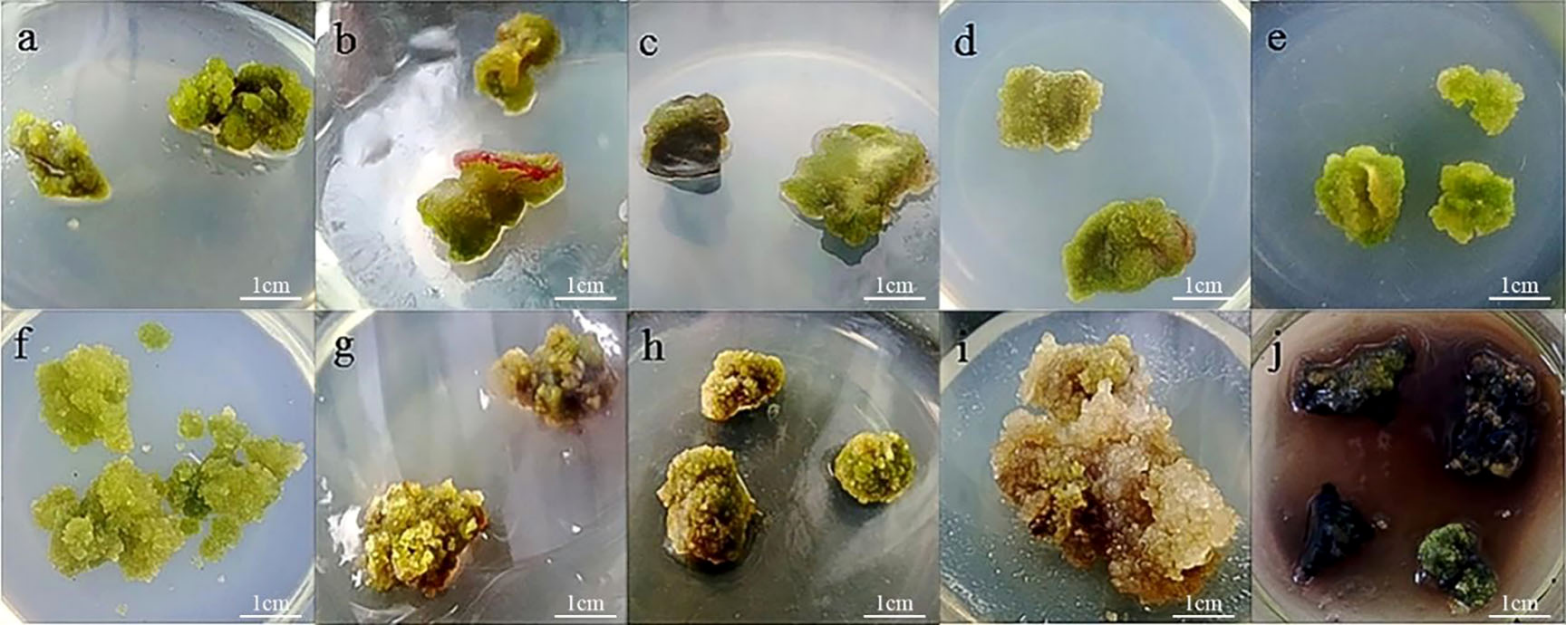
Callus induction and proliferation of *P. lactiflora*. **(A)** callus from cotyledon, **(B)** callus from stem, **(C)** callus from leave, **(D)** callus induction on MS medium containing 1.0 mg·L^-1^ 2,4-D, 0.5 mg·L^-1^ NAA and 0.5 mg·L^-1^ TDZ after culture for 30 days, **(E)** callus induction on MS medium containing 0.5 mg·L^-1^ 2,4-D, 1.0 mg·L^-1^ NAA and 0.5 mg·L^-1^ TDZ after culture for 30 days, **(F)** proliferation of callus from cotyledon, **(G)** proliferation of callus from stem, **(H)** proliferation of callus from leave, **(I)** waterlogged callus, **(J)** browned callus.

**Figure 2:**
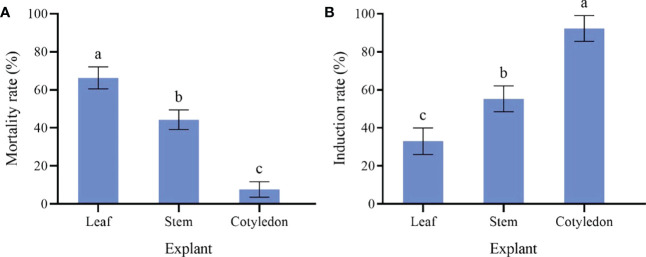
Effect of different explant of sterile seedling on *P. lactiflora* callus induction. **(A)** mortality rate of callus induced by different explants, **(B)** induction rate of callus induced by different explants, the different letters within a column marked significant differences (p < 0.05).

The cotyledons are the most likely to produce callus (**Figure 1A**). Cotyledons became thicker after 3 d of culture, and light green callus was generated after 10 d. It had the highest induction rate (92.33%) and the lowest mortality rate (7.67%) (**Figure 2**). After 10 d of culture, the stem generated green callus gradually, with induction rate and mortality rate were 55.33% and 44.33%, respectively (**Figures 1B** and**2**). However, leaves were not suitable for inducing callus. Although some leaves could generate calluses after 10 d of culture, most leaves died after 20 d with the highest mortality rate (66.33%) and the lowest induction rate (33.00%) (**Figures 1C** and**2**). Thus, the optimal explant for callus induction is the cotyledon.

### Effect of 2, 4-D, NAA, TDZ and 6-BA on callus induction

Auxin had an important influence on the induction rate of callus. After 20 d of culture on MS medium containing 2,4-D, the callus produced and grew slowly. With the increase of 2,4-D concentration, the induction rate increased gradually. The highest value was 57.78% when treated with 1.0 mg·L^-1^ 2,4-D. The calluses in Group 1 were in a better growth state and presented green and full of vitality (**Table 1**). While on MS medium containing NAA, the cotyledon generated callus only after 7 d of culture. After 20 d, the whole cotyledon became callus. When the concentration of NAA was 0.5 mg·L^-1^, the callus in Group 2 induction rate reached the maximum (**Table 1**). However, the callus presented as unhealthy, waterlogged, yellow-white and loose. Some callus stopped growing and even died due to browning. Therefore, 2,4-D was beneficial to callus growth, while NAA could decrease the time required for callus induction.

Cytokinin is also indispensable in the process of inducing callus. Cytokinin promotes cell division and differentiation. An appropriate concentration of cytokinin and auxin will effectively improve the induction efficiency of callus. Based on the results described, 1.0 mg·L^-1^ 2,4-D was added to MS medium. At the same time, different concentrations of 6-BA or TDZ were added. After adding cytokinin, the whole cotyledon became calluses for an average culture of 15 d, and the callus growth state was effectively improved. High or low TDZ concentration was not conducive to the callus induction. The highest rate of callus induction (67.78%) was in Group 3, which was observed from the 1.0 mg·L^-1^ TDZ (**Table 1**). The callus was fresh green and loose. Although there was a small amount of browning, it was effectively improved by transferring to a fresh medium. It could be seen from the fourth group that 1.0 mg·L^-1^ 6-BA had the highest induction efficiency (**Table 1**). The callus presented yellow-green and dense. After 40 d, the callus browning occurred and the browning degree did not decrease after transferring to a fresh medium. Thus, TDZ was more effective on callus induction than 6-BA.

The presence of 2,4-D was important to ensure callus growth state, while NAA was important for the velocity of callus induction. We found that the combination of NAA, 2,4-D and TDZ was more conducive to the callus induction. Meanwhile, the callus presented fresh green, dense and full of vitality (**Table 1**). On the average culture of 10 d, the whole cotyledon became callus. The highest induction rate (86.67%) was observed on MS medium supplemented with 1.0 mg·L^-1^2,4-D, 0.5 mg·L^-1^NAA and 0.5 mg·L^-1^ TDZ or MS medium supplemented with 0.5 mg·L^-1^ 2,4-D, 1.0 mg·L^-1^ NAA and 0.5 mg·L^-1^ TDZ. The latter was in a better growth state and presented less browning after culture (**Figure 1D** and **Figure 1E**). Therefore, the optimal medium for callus induction was MS medium containing 0.5 mg·L^-1^ 2,4-D, 1.0 mg·L^-1^ NAA and 0.5 mg·L^-1^ TDZ.

### Effect of different explants on callus proliferation

**Figure 3:**
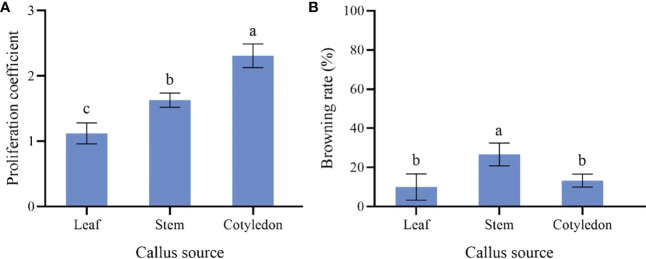
Effect of different explants on *P. lactiflora* callus proliferation. **(A)** proliferation coefficient of callus induced by different explants, **(B)** browning rate of callus induced by different explants, the different letters within a column marked significant differences (p < 0.05).

The proliferation of callus from the cotyledon was fast, which presented emerald green, tight and uniform texture (**Figure 1F**). The highest proliferation coefficient (2.31) was observed on callus from the cotyledon and the browning rate was 13.33% (**Figure 3**). Callus induced from the stem had the lowest proliferation coefficient (1.12) and poor growth status. Reduced fullness and soft texture were observed during the culture (**Figure 1G**), with the highest browning rate (26.67%) and a serious browning degree (**Figure 3**). The proliferation coefficient was 1.63 of leaf callus. Its texture was compact (**Figure 1H**) and the proliferation rate was slower, while the browning rate was lower (10.00%). Therefore, callus from the cotyledon was the best choice for callus proliferation.

### Effects of NAA, 2, 4-D and TDZ on callus proliferation

Considering that the effect of 6-BA on callus was not as good as TDZ, we did not use 6-BA in this experiment. When the concentration of TDZ was constant, 2,4-D and NAA had different effects on callus proliferation. Different concentrations of 2,4-D resulted in no significant difference in proliferation coefficient, while the browning rate increased gradually with the increase of 2,4-D concentration (**Table 2**, Group 1). When the concentration of TDZ was the constant, high or low concentration of NAA was not conducive to the callus proliferation. The maximum proliferation coefficient (1.68) and lowest browning rate (23.33%) were observed when 0.5 mg·L^-1^ NAA was applied (**Table 2**, Group 2). The callus presented yellow-green and loose with the supplement of 2,4-D, while the callus showed different degrees of green, full and loose texture due to the NAA supplement. On these two mediums, the callus will gradually brown from the inside to the outside after a culture of 30 d. Although browning will be effectively alleviated by transfer, the callus aged and even produced white aerial roots. According to this study, 2,4-D was better than NAA on improving callus proliferation coefficient, and the browning rate was lower (**Table 2**). As for the growth state, NAA had a better effect.

Based on the results of callus proliferation, NAA, 2,4-D and TDZ were combined to improve the callus proliferation and this brought a better effect (**Table 2**, Group 3). After being transferred to a proliferation medium for 20 d, the callus proliferated vigorously and presented a green, full and loose growth state. However, browning occurred at varying degrees. The callus proliferation coefficient reached the highest value (3.21) on the MS medium containing 0.5 mg·L^-1^NAA, 1.0 mg·L^-1^2,4-D and 0.5 mg·L^-1^ TDZ, followed by the 2.80, which was observed on the MS medium containing 1.0 mg·L^-1^ NAA, 0.5 mg·L^-1^ 2,4-D and 0.5 mg·L^-1^ TDZ. Their browning rates were 33.33% and 41.11%, respectively. Therefore, the optimal medium for callus proliferation was MS medium containing 0.5 mg·L^-1^ NAA, 1.0 mg·L^-1^ 2,4-D and 0.5 mg·L^-1^ TDZ.

### Effect of different kanamycin and cefotaxime concentration on *P. lactiflora* callus growth

**Figure 4:**
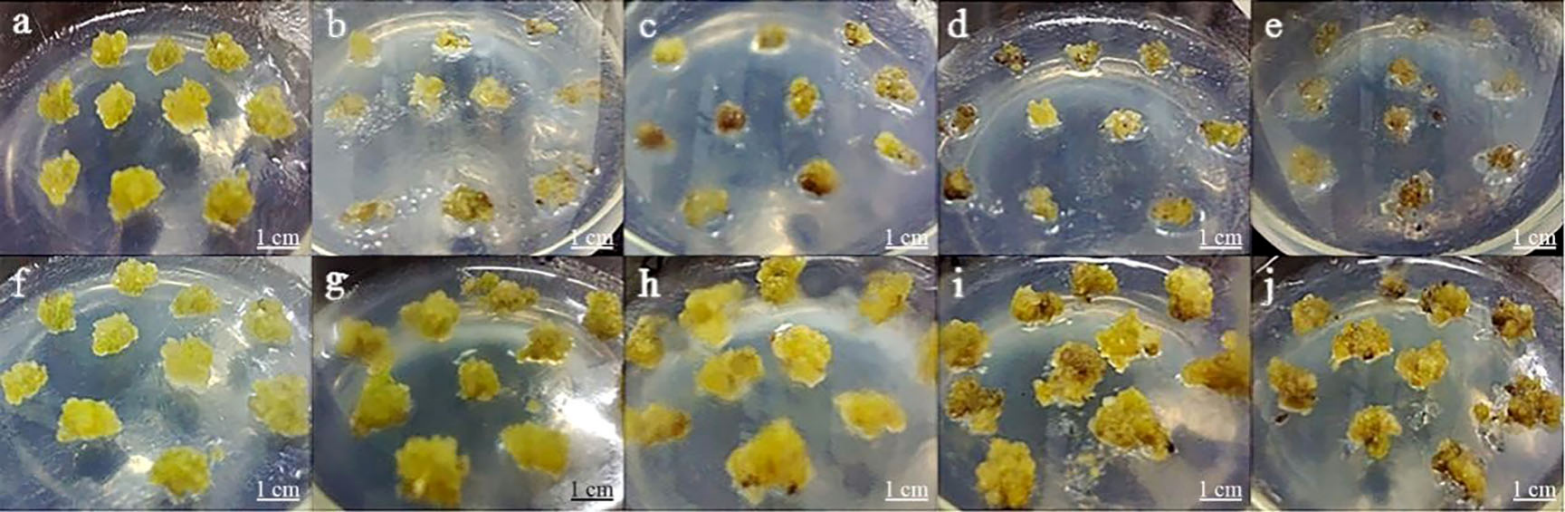
Antibiotic sensitivity assay of *P. lactiflora* callus. **(A-E)** kanamycin concentrations were 0, 75, 100, 125 and 150 mg·L^-1^, respectively. **(F-J)** cefotaxime concentrations were 0, 100, 200, 300, and 400 mg·L^-1^, respectively.

In this assay, the growth state of *P. lactiflora* callus was observed on a proliferation medium containing various concentrations of Kan or Cef (**Figure 4**). The callus was more sensitive to Kan. 75~150 mg·L^-1^ of Kan could strongly inhibit the growth of *P. lactiflora* callus. The higher concentration led to the lower proliferation rate and more serious browning of calluses, and even dead calluses (**Table 3**). According to the growth state, 125 mg·L^-1^ Kan was chosen to screen transformed callus of *P. lactiflora*.

**Table 3:** Kanamycin effects on *P. lactiflora* callus proliferation.

Concentration of kanamycin (mg·L^-1^)	0	75	100	125	150
Proliferation rate (%)	100 ± 0.01a	10.8 ± 0.58b	5.4 ± 0.79bc	0.00 ± 0.00c	0.00 ± 0.00c
Browning rate (%)	18,97 ± 2.33e	26.64 ± 1.86cd	27.68 ± 1.92bc	29.06 ± 1.32b	32.7 ± 1.47a

The values represented mean ± SD, and different letters marked significant differences (p < 0.05).

Cef could inhibit the growth of *P. lactiflora* callus. The callus was less sensitive to Cef compared with Kan. Both 100, 200 and 300 mg·L^-1^ of Cef had an insignificant impact on the growth and proliferation of callus, while 400 mg·L^-1^ Cef could inhibit the callus obviously. Each callus presented browning. Combining its effect on *Agrobacterium* growth (**Table 4**), the optimal concentration of Cef might be 200 mg·L^-1^.

**Table 4:** Cefotaxime effects on *Agrobacterium* growth.

Concentration of cefotaxime (mg·L^-1^)	0	100	200	300	400
Cell density of *Agrobacterium* (OD_600_)	1.17 ± 0.01a	0.12 ± 0.01b	0.00 ± 0.00c	0.00 ± 0.00c	0.00 ± 0.00c

The values represented mean ± SD, and different letters marked significant differences (p < 0.05).

### Inhibitory effect of cefotaxime on *Agrobacterium* tumefaciens

For exploring the inhibitory effect of cefotaxime on *Agrobacterium tumefaciens*, we carried out the inhibition test of cefotaxime on *Agrobacterium*. The results showed that the OD_600_ value could not be detected when the concentration of cefotaxime was 200, 300 and 400 mg·L^-1^ (**Table 4**). Considering the effect of Cef on the callus growth of *P. lactiflora*, the optimal concentration of Cef was 200 mg·L^-1^.

### Transformation and obtaining of transgenic callus

**Figure 5:**
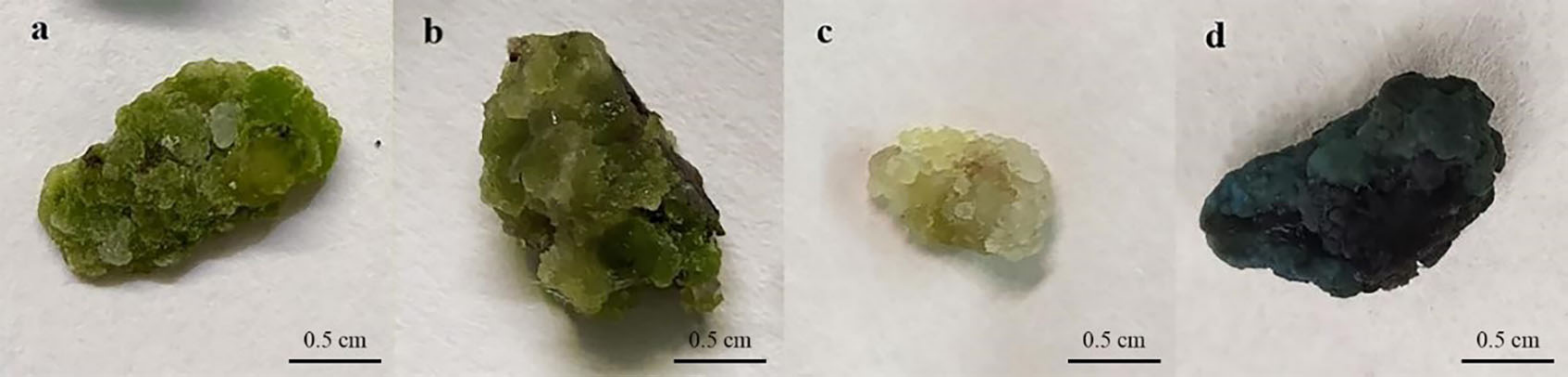
Genetic transformation of *P. lactiflora* callus by *Agrobacterium*-mediated transformation. **(A)** callus before *Agrobacterium* infection, **(B)** callus after *Agrobacterium* infection, **(C)** GUS expression in control callus, **(D)** GUS expression in transgenic callus.

In order to establish genetic transformation system and obtain transgenic callus, *Agrobacterium* strain EHA105 harboring the binary vector pBI121-GUS was used to infect callus. Various combinations of cell densities of *Agrobacterium* suspension at OD_600_ (0.2, 0.4, 0.6, 0.8 and 1.0), infection duration (10, 15, 20, 25 and 30 min) and co-culture duration (0, 1, 2, 3 and 4 d) were carried out to select the optimum transgenic system. Then, the GUS staining was used for the identification of transgenic callus after selection culture for 49 d (**Figure 5**).

**Figure 6:**
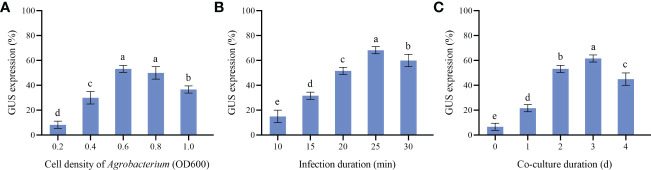
Parameters affecting transformation efficiency. **(A)** GUS expression at different *Agrobacterium* cell density, **(B)** GUS expression at different Infection duration, **(C)** GUS expression at different Co-culture duration, the different letters within a column marked significant differences (p < 0.05).

Calluses induced by cotyledons with good and consistent growth were selected for *Agrobacterium* transformation in this experiment. *Agrobacterium* cell densities, infection and co-culture duration are key transformation factors directly affecting genetic transformation. Our results showed that the effects of different *Agrobacterium* cell densities were quite different (**Figure 6**). High or low cell densities were not conducive to the genetic transformation of *P. lactiflora* callus. When OD_600_ was 0.6, the callus attained the maximum GUS expression rate (53.33%). In the same way, we also found that 25 min of infection and 3 d of co-culture led to the highest GUS expression rate, with 68.33% and 61.67%, respectively. Therefore, OD_600_ = 0.6, 25 min for infection and co-culture for 3 d were chosen as the optimal *Agrobacterium* transformation conditions.

### Molecular identification of transformants

**Figure 7:**
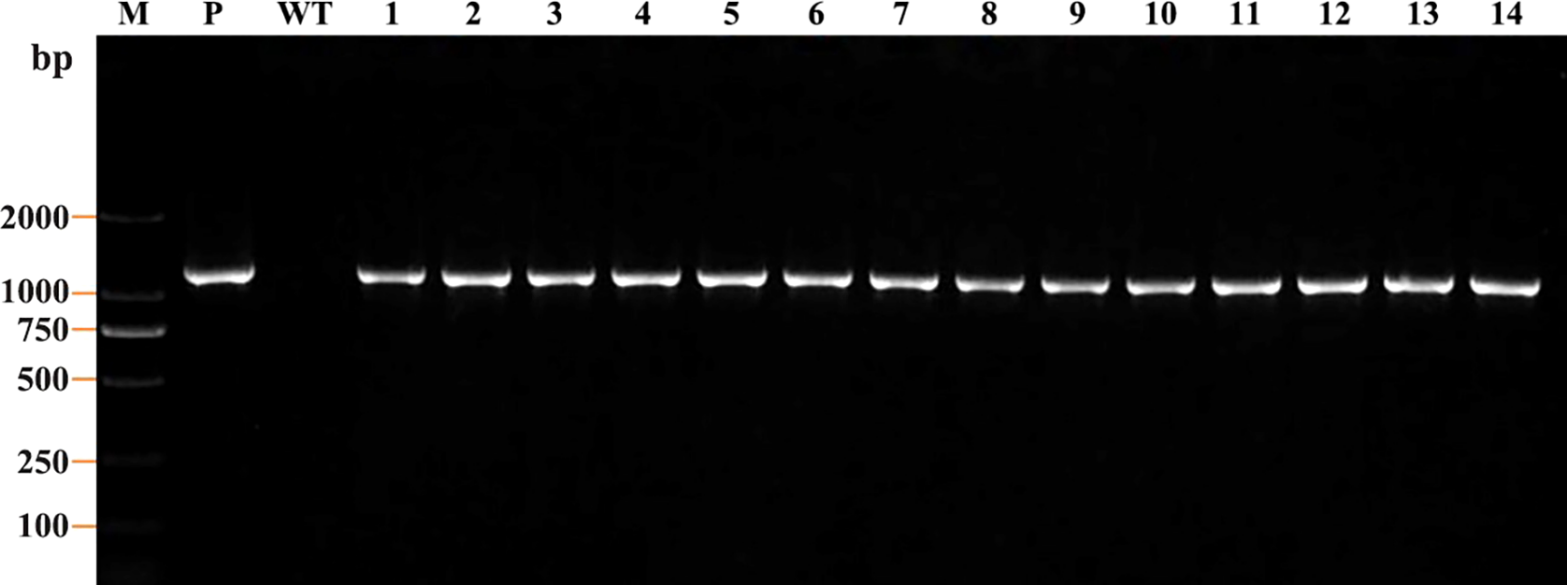
PCR analysis of the GUS-positive resistant callus of *P. lactiflora*. PCR product in 1182 bp was observed in each positive callus except wild-type with *GUS* gene-specific primer. Lane M, DL2000 DNA marker; lane P, plasmid control; lane WT, wild-type callus; lanes 1-14, different GUS-positive callus.

**Figure 8:**
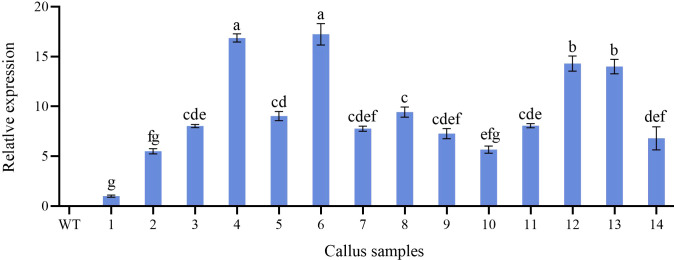
Relative expression level of *GUS* gene in non-transformed and transgenic callus. WT, non-transformed callus; 1-14, different transgenic callus, the different letters within a column marked significant differences (p < 0.05).

Further molecular identification of the resistant calluses was confirmed by PCR and RT-qPCR. GUS-positive resistant calluses were selected randomly and confirmed by PCR and RT-qPCR. In PCR validation test, plasmid pBI121 was used as the positive control and non-transgenic callus DNA was used as the negative control. The presence of amplified fragments in GUS-positive resistant calluses samples and pBI121 plasmid confirmed the presence and integration of the *GUS* gene in the *P. lactiflora* genome. Non-transgenic callus genomic DNA did not show any amplified fragments (**Figure 7**). In order to further identify the transgenic callus at the transcriptional level, RT-qPCR was used to detect the expression level of the *GUS* gene in non-transgenic callus and GUS-positive resistant calluses. The results showed that the *GUS* gene expression was not detected in the non-transgenic callus, but it was detected in GUS-positive resistant calluses (**Figure 8**).

## Discussion

Plant callus is considered to have the potential to be used in rapid mass propagation of plants because of its advantages of easy access, strong ability of proliferation and differentiation (Wang et al., 2021b). However, callus induction and proliferation are distinct in different plant species (Xu et al., 2017). The success of callus induction is strongly related to the choice of the explant. Explant source and growth physiological state will directly affect the process of callus induction (Guo and Jeong, 2021). Seeds, cotyledons, stems, leaves, anthers, short limbs and even young fruits are usually used as explants for callus induction (Singh et al., 2015; Din et al., 2016; Bai et al., 2019). In fact, there have been many studies on callus induction in herbaceous peony, in which explants selection has been generally concerned (Shen et al., 2012). When the underground buds of *P. lacactiflora* were used as explants, the buds developed well and had strong ability to differentiate and proliferate *in vitro* (Tian et al., 2010). Orlikowska et al. used petioles, leaves and young stems of *Paeonia loniflorica* as explants for induction experiments. Their results showed that whether these explants could induce callus successfully depended on their culture conditions in the first 1.5 months, and the young stems were considered to have the most differentiation potential (Orlikowska et al.,1998). For wild herbaceous peony, the callus induction from vegetative organs was not only limited by season, but also complicated and ineffective in sterilization (Shen et al., 2012). In the present study, we further optimized the selection of explants based on the experience of previous studies. We induced callus using different parts of sterile seedlings obtained from cultivated seeds. The reason we did this is that seeds are easy to preserve and obtain, ensuring that explants are available regardless of time and season. In addition, the obtained sterile seedlings don’t need to be sterilized, which could improve the induction efficiency. In our study, the most suitable explant for callus induction was sterile seedling cotyledon (**Figure 2**), and the order of proliferation ability of callus from different explants was cotyledon > leaf > stem (**Figure 3**). Therefore, cotyledons obtained from seed culture was most suitable for callus induction and proliferation. The reason for this result, we believe, is that cotyledon emerges at the early stage of growth and development, and the meristem cells are more active (Hu et al., 2015). Just because of this reason, we further speculated that the callus induced by cotyledon had strong growth vigor and high proliferation efficiency. Stem and leaf are at the mid-growth stage. Although their cells are totipotent, the cells of plant organs have already differentiated. So, the dedifferentiation ability of stem and leaf is relatively reduced, which may cause low efficiency in the callus induction and proliferation.

Adding plant growth regulators to the basal medium will make different effects on callus induction and proliferation (Kotb et al., 2020). Many previous studies have proved that PGRs are very important for plant callus induction and proliferation. The combination of auxin and cytokinin could improve the induction rate of callus, and the induction rate was higher when the ratio of cytokinin to auxin was between 1:1.4 and 1:5 (Shen et al., 2012). TDZ and 6-BA are both important cytokinins and play a significant role in the induction and proliferation of plant callus (Roy et al., 2012). TDZ has stronger cytokinin activity, and is superior to 6-BA in both callus and branch proliferation (Zhao et al., 2017). Similarly, our results proved that TDZ was also superior to 6-BA in callus induction of *P.lactiflora* (**Table 1**). In this study, on the basis of previous studies, we further optimized the components of the culture medium for callus induction from *P. lactiflora*.The results showed that two auxins, 2,4-D and NAA, affected *P. lactiflora* callus induction significantly. On 2,4-D supplemented medium, the induced callus was bright green, dense and high viable, while the induced callus was loose, water-stained, low viable and browning occurred on NAA supplemented medium (**Table 1**). Therefore, we believe that 2,4-D had a better effect on callus induction than NAA, which was consistent with the previous study (Wang et al., 2010). Our results also show that when combined with TDZ in the process of proliferation, the callus proliferation coefficient was greater in a medium containing 2,4-D than that in a medium containing NAA at the same concentration. The combination of 2,4-D, NAA and TDZ were more effective in both the callus induction and proliferation process, maintaining a higher induction rate and proliferation coefficient as well as a better callus growth state. These results again indicate that 2, 4-D, NAA and TDZ play an important role in callus proliferation.In the course of our experiments, we unexpectedly discovered some preliminary methods that may reduce the browning in *P. lactiflora* tissue culture. Browning is a common phenomenon in plant tissue culture and has always been a big hurdle in the tissue culture of *P. lactiflora* (Gao et al., 2020). The explants release brown substances or phenolics into the medium in the course of plant tissue culture, which can inhibit the normal growth and differentiation of cell tissues, and even lead to death (Shen et al., 2012). Plant growth regulators have a certain effect on the browning of callus (Shen et al., 2012). In our present study, in the medium supplemented with 2,4-D, the callus was lightly browned and browning could be effectively suppressed by transferring to a new medium, while callus browning increased in the NAA-added medium. When the ratio of auxin and cytokinin varied from 1:1 to 2:1, the browning degree was lighter. Moreover, frequent transfer could slightly reduce the degree of browning but the callus gradually stopped growing after multiple transfers. Our findings may be helpful in alleviating the browning of *P. lactiflora* tissue culture, but the novel methods and mechanisms of browning prevention have yet to be obtained.

During genetic transformation, the main role of antibiotics such as Kan and Cef is to screen resistant plants or to inhibit the overgrowth of *Agrobacterium*. Previous studies have reported the optimal concentration of Kan (5-100 mg·L^-1^) and Cef (50-400 mg·L^-1^) in plants of different genera (Matsuda et al., 2005; Freiman et al., 2012; Yang et al., 2017). These studies indicated that different genotypes have different degrees of sensitivity to antibiotics, suggesting the importance of screening antibiotic concentration during transformation. In our study, we found that 125 mg·L^-1^ Kan could completely inhibit the growth of callus, and 200 mg·L^-1^ Cef was the optimal concentration to inhibit the growth of *Agrobacterium* without affecting the growth of callus. (**Table 4** and **Figure 4**). We believe that under the action of these two antibiotics, not only positive transgenic callus can be obtained, but also the growth of *Agrobacterium* can be completely inhibited, so as to avoid the pollution caused by *Agrobacterium* attachment during molecular identification.

The *Agrobacterium*-mediated genetic transformation is the most widely used transformation method (Liu et al., 2021). However, there were few applications on *P. lactiflora*. Transformation receptor and condition are key factors affecting genetic transformation (Shen et al., 2012). Plant callus is very suitable as transformation receptor (Liu et al., 2021). It is generally believed that the pre-culture and wound of explants before transformation can improve the transformation rate of exogenous genes (Wu et al., 2014; Yan et al., 2019). Nevertheless, callus can be used as acceptor material without considering these steps (Sedaghati et al., 2018). *Agrobacterium* cell density, infection time and co-culture time have important effects on genetic transformation efficiency, and optimal parameters vary depending on the plant materials and/or acceptor types (Sedaghati et al., 2018; Yan et al., 2019). Low cell density may not provide sufficient bacteria and short periods are not conducive to establish contact between the acceptor material and *Agrobacterium*. High cell density or long co-culture time will easily cause contamination (Yan et al., 2019). Extreme cell density and infection time affect the transformation efficiency because of the *Agrobacterium* toxicosis or incomplete contact with the surface of callus (Ahmed et al., 2015). Short co-culture time may lead to incomplete T-DNA transfer process. Conversely, long co-culture time will lead to the excessive multiplication of *Agrobacterium*, which poisons the callus (Wu et al., 2015). In order to determine the optimal transformation conditions for *Agrobacterium*-mediated genetic transformation and whether the target gene can be effectively transferred into peony callus, we used GUS staining experiment to observe the transformation efficiency of the target gene. From the results, we can also see that different amounts of GUS chromogenesis were observed under various transformation conditions designed by us. *Agrobacterium* cell density at 0.6 OD_600_, 25 min of infection and 3 d of co-culture showed high GUS staining positive rate during the screening process (**Figure 6**). PCR and RT-qPCR are commonly used to detect transgenic plants (Rajeevkumar et al., 2015; Wu et al., 2019; Wang et al., 2021b). As can be seen from PCR results, clear and bright amplified fragments were observed in 14 GUS-positive samples randomly selected, and the length of the fragments was consistent with the target gene (**Figure 7**). This indicates that our method can successfully transfer the target gene into the callus of *P. lactiflora*. RT-qPCR results also showed that GUS gene expression was detected in all GUS-positive resistant calluses compared with the non-transgenic callus, although GUS gene expression was slightly different in different GUS-positive resistant calluses (**Figure 8**). However, from the results, the relative expression levels of target genes in most samples are relatively stable, which indicates that our method is feasible.

## Conclusion

In conclusion, this study optimized and established an efficient method for callus induction and proliferation of *P. lactiflora*. Based on this method, cotyledons were used as explants to induce and proliferate high-quality callus, and then an efficient transgenic method using callus of *P. lactiflora* was established through *Agrobacterium* mediated genetic transformation. Our results provide theoretical basis and technical support for the establishment of a complete and stable genetic transformation system of herbaceous peony and further understanding of the molecular and genetic mechanisms related to herbaceous peony.

## Data availability statement

The datasets presented in this study can be found in online repositories. The names of the repository/repositories and accession number(s) can be found in the article/Supplementary Material.

## Author contributions

XS, SD and XL designed the project. WC, QP, XL, SD and RF completed the callus induction and proliferation experiment. SD, RX and XL completed *Agrobacterium*-mediated transformation conditions screening experiment. XL and RF were involved in GUS staining experiment. This article was written by SD, XL and SG. All authors contributed to the article and approved the submitted version.
